# Pulmonary Rehabilitation in Patients with Chronic Lung Impairment from Pulmonary Tuberculosis

**DOI:** 10.7759/cureus.3664

**Published:** 2018-11-30

**Authors:** Seema K Singh, Ashutossh Naaraayan, Prakash Acharya, Balakrishnan Menon, Vishal Bansal, Stephen Jesmajian

**Affiliations:** 1 Internal Medicine, Montefiore New Rochelle Hospital, Albert Einstein College of Medicine, New Rochelle, USA; 2 Pulmonary Medicine, Vallabhbhai Patel Chest Institute, University of Delhi, New Delhi, IND; 3 Physiology, Vallabhbhai Patel Chest Institute, University of Delhi, New Delhi, IND

**Keywords:** health related quality of life, pulmonary function tests, pulmonary rehabilitation, pulmonary tuberculosis, six-minute walk distance

## Abstract

Setting

Our study was conducted at a tertiary care center for respiratory illnesses (Viswanathan Chest Hospital, Vallabhbhai Patel Chest Institute (VPCI), University of Delhi, Delhi, India). Patients were enrolled in the study from the outpatient clinic.

Objective

To assess the effects of pulmonary rehabilitation (PR) in patients with chronic lung impairment from previously treated tuberculosis (CLIPTB), on exercise capacity (six-minute walk distance), pulmonary function tests, quality of life and markers of systemic inflammation.

Design

Prospective cohort study including 29 patients who had finished anti-tubercular therapy and currently had symptoms of dyspnea with or without cough secondary to CLIPTB.

Result

Significant improvement in six-minute walk distance (488 meters at baseline vs 526 meters post PR intervention, p-value 0.033) and chronic respiratory questionnaire score (17.21 at baseline vs 18.96 post PR intervention, p-value 0.025) with pulmonary rehabilitation was noted. Pulmonary function tests, inflammatory markers and mid-thigh muscle mass trended towards improvement with pulmonary rehabilitation but were not statistically significant.

Conclusion

Our study shows that pulmonary rehabilitation is an effective intervention in post-tuberculosis patients and should be recommended.

## Introduction

Tuberculosis (TB) is the ninth leading cause of death worldwide and the leading cause from a single infectious agent, ranking above human immunodeficiency virus (HIV)/acquired immunodeficiency syndrome (AIDS). In 2016, there were an estimated 1.3 million TB deaths among HIV-negative and an additional 374,000 deaths among HIV-positive people. An estimated 10.4 million people fell ill with TB in 2016 — 90% were adults, 65% were male, 10% were people living with HIV (74% in Africa) and 56% were from five countries: India, Indonesia, China, Philippines and Pakistan [1]. TB is a major public health problem in India. India accounts for ~27% of the global TB incident cases. Each year an estimated 2.7 million people in India develop TB, although the incident-reported cases were about ~1.9 million in 2016. Approximately 84% of reported cases are of pulmonary origin. It is estimated that around 435,000 Indians died due to TB in 2016 [2].

The End TB strategy of the World Health Organization (WHO) 2015 has set up goals to end the TB epidemic [3]. The goal is to achieve a 95% reduction in TB deaths and a 90% reduction in TB incidence rate by 2035 compared to 2015. WHO recommends focusing on early detection, diagnosis and standardized supervised treatment of TB patients. Emphasis is also placed on monitoring and evaluating the impact of this strategy. However, there is no emphasis placed on following up on the chronic morbidity and mortality that may ensue after successful treatment of TB.

Long-term follow-up studies have found that the risk of death in patients successfully completing anti-tuberculosis treatment is high, with mortality rates consistently above those observed in the general population [4]. The cause of increased mortality is likely multifactorial from comorbidities such as human immunodeficiency viral infection, diabetes mellitus as well as superinfections. Several studies have demonstrated abnormal lung functions and chronic lung impairment in previously treated TB patients and this likely contributes to increased mortality as well [5,6]. Despite adequate treatment, pulmonary tuberculosis (pTB) may lead to chronic bronchial and parenchymal structural changes, including bronchiectasis and emphysematous changes [7]. These chronic anatomic, physiologic and symptomatic (dyspnea/cough) sequelae from previously treated pTB are being referred to as chronic lung impairment from previously treated tuberculosis (CLIPTB) in our study.

Pulmonary rehabilitation (PR) has been recognized as a core component for the management of patients with chronic respiratory diseases including chronic obstructive pulmonary disease (COPD), bronchiectasis, interstitial lung disease (ILD) among others [8]. PR has been shown to improve symptoms, exercise tolerance and health-related quality of life (HRQL) in these patients [8]. PR has been found to be one of the most cost-effective treatments for COPD, falling behind only influenza vaccination and pharmacotherapy for smoking cessation and faring better than all the inhaled treatments available for COPD [9,10]. There has been a growing body of literature describing benefits of PR in patients with CLIPTB [11,12]. We conducted our study to assess the effects of PR in patients with CLIPTB, not just on the quality of life and functional status, but on markers of systemic inflammation as well.

## Materials and methods

**Figure 1:**
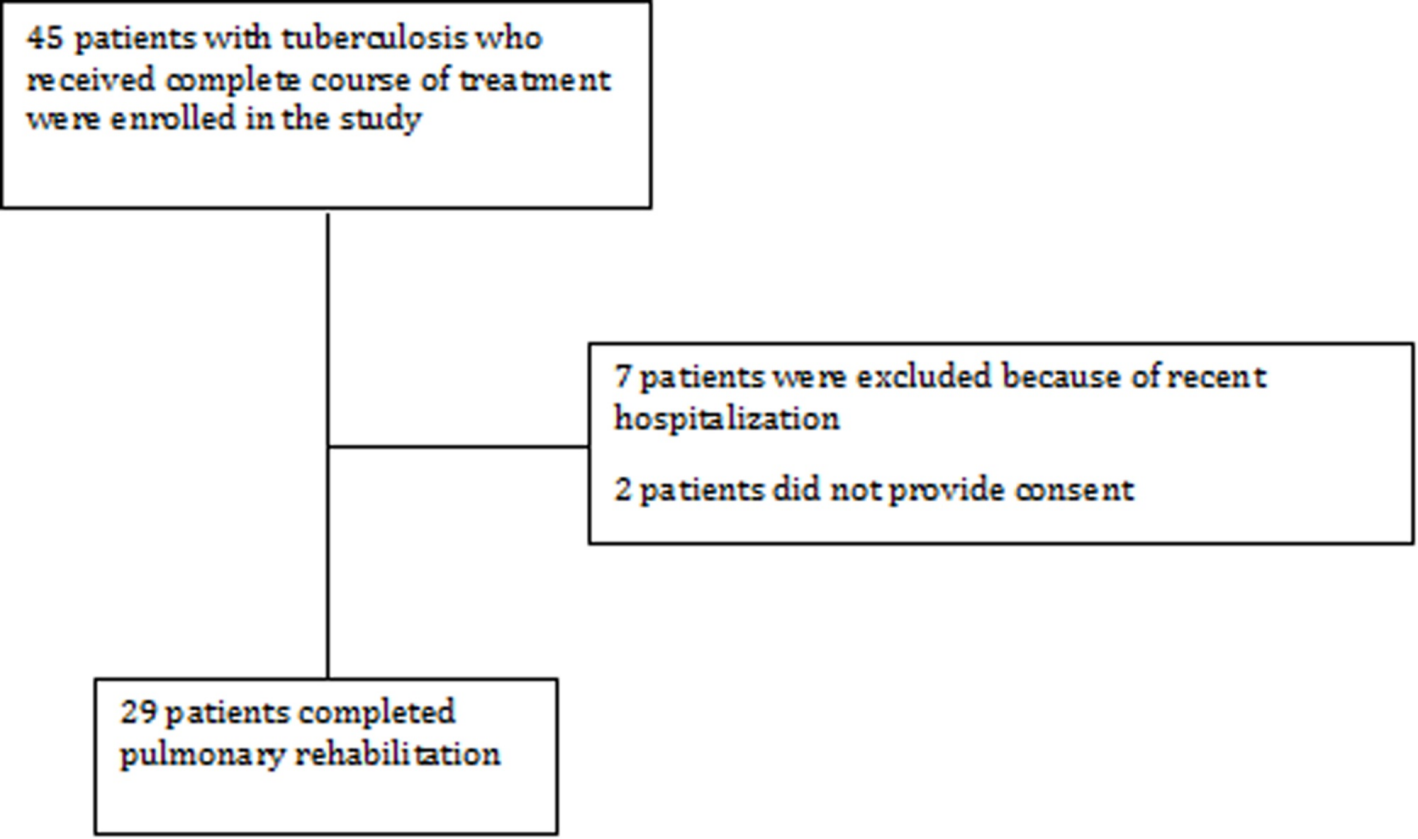
Selection of patients.

This is a prospective cohort study performed at Viswanathan Chest Hospital, Vallabhbhai Patel Chest Institute (VPCI), University of Delhi, Delhi, India. Forty-five CLIPTB patients, with symptoms of dyspnea with or without cough, who were being followed up in the outpatient clinic were enrolled between June 2011 and May 2012. Patients between the ages 18 and 50 years, who were diagnosed with pTB, treated with a complete course of anti-tubercular therapy (ATT) within the past 10 years and were currently sputum smear and culture negative, were included. Patients with positive sputum acid fast bacteria (AFB) or culture, concomitant asthma or COPD, recent hospitalization or steroid use and physical disability not permitting PR were excluded from the study. Current smokers and pregnant/lactating women were also excluded. Twenty-nine patients were included in the final analysis (Figure 1).

In the initial run-in phase of the study, we administered formoterol and salbutamol inhalers to our cohort for a period of eight weeks. Baseline investigations were done at the end of the run-in period (Table 1).

**Table 1:** Baseline characteristics of the study cohort. CRQ: Chronic respiratory disease questionnaire; FEV1: Forced expiratory volume in 1 second; FVC: Forced vital capacity; hsCRP: high sensitivity C-reactive protein; HRQL: Health related quality of life; MMRC: Modified Medical Research Council dyspnea scale; MTCSA_CT_: Mid-thigh cross section area by computed tomography; SD: Standard deviation; TGF-β1: Transforming growth factor-β1.

Characteristics	Findings
Age, median (years)	48
Gender n (%)	
Male	11 (37.94)
Female	18 (62.06)
Mean body mass index (kg/m^2^)	22
Prior history of smoking n (%)	5 (17.24)
Duration of disease, median (years)	8
Use of supplemental oxygen	5 (17.24)
HRQL measures	
MMRC grade, mean (SD)	2.1 (0.72)
Chronic respiratory questionnaire (CRQ), mean (SD)	17.21 (2.86)
Exercise capacity, median (SD)	
Six-minute walk distance (meters) (SD)	488 (110.13)
Pulmonary function test, mean (SD)	
FEV1 (liters)	1.02 (0.32)
FVC (liters)	1.76 (0.54)
FEV1/FVC	60.98 (16.37)
Markers of inflammation, median (SD)	
hs-CRP (μg/L)	4.3 (3.02)
TGF-β1 (ng/ml)	1.3 (3.55)
Markers of muscle mass, median (SD)	
MTCSA_CT _(mm^2^)	9222.3 (1342.06)

All the patients received PR for a period of eight weeks. The PR program included a minimum of 90 minutes of supervised exercise training for lower and upper limbs, three days a week. Lower limb training included leg-ergometry and treadmill walking. Training of the upper limbs included arm-ergometry and free weights. Simultaneous upper and lower limb training was performed on semi-recumbent whole body exerciser. Exercise intensity during each session was incremental and graded according to symptom tolerance. Patients also attended educational sessions on breathing exercises, energy conservation, lung health, medications and stress management. At the end of eight weeks of PR program, investigations were repeated. These results were compared to the baseline.

PR was the main exposure and change in six-minute walk distance (6-MWD) was the primary outcome. 6-MWD was performed as per the American Thoracic Society guidelines as described in Supplement 2. Changes in inflammatory markers [high sensitivity C-reactive protein (hs-CRP) and transforming growth factor-β1 (TGF-β1)], pulmonary function tests (PFTs) [forced expiratory volume in one second (FEV1), forced vital capacity (FVC) and FEV1/FVC ratio)], mid-thigh cross sectional area by computed tomography (MTCSA_CT_) and HRQL measures [modified medical research council scale (MMRC) and chronic respiratory disease questionnaire (CRQ)] were the secondary outcomes. Differences in outcomes before and after PR were compared using Mann-Whitney U Test/Wilcoxon rank-sum test and Chi square test. All reported statistical tests were two-sided and p-value < 0.05 was considered statistically significant. All the analysis was performed using Stata 14.0 (Stata Corp, College Station, TX).

The study was conducted after getting approval from the ethical committee of VPCI. Written informed consent was obtained from each patient prior to admission to the study.

## Results

Of the 45 patients enrolled in the study, 29 were included in the analysis. The median age of the population was 48 years and 62% of the patients were females. The median time since tuberculosis treatment was eight years and 17% of the patients had a history of prior smoking. At baseline median 6-MWD was 488 meters, median hs-CRP levels were 4.3 mcl/liter and median TGF-β1 levels were 1.3 ng/ml. Median MTCSA_CT_ was 9222.3 mm^2^ at baseline. Baseline mean CRQ score was 17.21, mean MMRC score was 2.1 and PFTs were as shown in Table 1.

After eight weeks of PR, there was no significant change in pulmonary function tests [FEV1 (1.023 liters vs 1.066 liters, p-value 0.623), FVC (1.76 liters vs 1.81 liters, p-value 0.74), FEV1/FVC (60.98 vs 60.55, p-value 0.92)] (Table 2).

**Table 2:** Outcomes with pulmonary rehabilitation. CRQ: Chronic respiratory disease questionnaire; FEV1: Forced expiratory volume in 1 second; FVC: Forced vital capacity; hsCRP: high sensitivity C-reactive protein; HRQL: Health related quality of life; MMRC: Modified Medical Research Council dyspnea scale; MTCSA_CT_: Mid-thigh cross section area by computed tomography; SD: Standard deviation; TGF-β1: Transforming growth factor-β1.

Outcomes	At baseline (Standard deviation)	At eight weeks (Standard deviation)	p-value
Pulmonary function tests (mean)			
FEV1	1.02 (0.32)	1.07 (0.33)	0.62
FVC	1.76 (0.54)	1.81 (0.56)	0.74
FEV1/FVC	60.98 (16.37)	60.55 (15.26)	0.92
Inflammatory markers (median)			
TGF-β1	1.3 (3.55)	1.43 (2.79)	0.9
hs-CRP	4.3 (3.02)	2.3 (3.39)	0.21
Muscle mass (median)			
MTCSA_CT_	9222.3 (1342.06)	9356.67 (1734.15)	0.33
Exercise capacity (median)			
Six-minute walk distance	488 (110.13)	526 (81.25)	0.033
HRQL measures			
MMRC	2.10 (0.72)	2 (0.71)	0.58
CRQ	17.21 (2.86)	18.96 (2.91)	0.02

**Figure 2:**
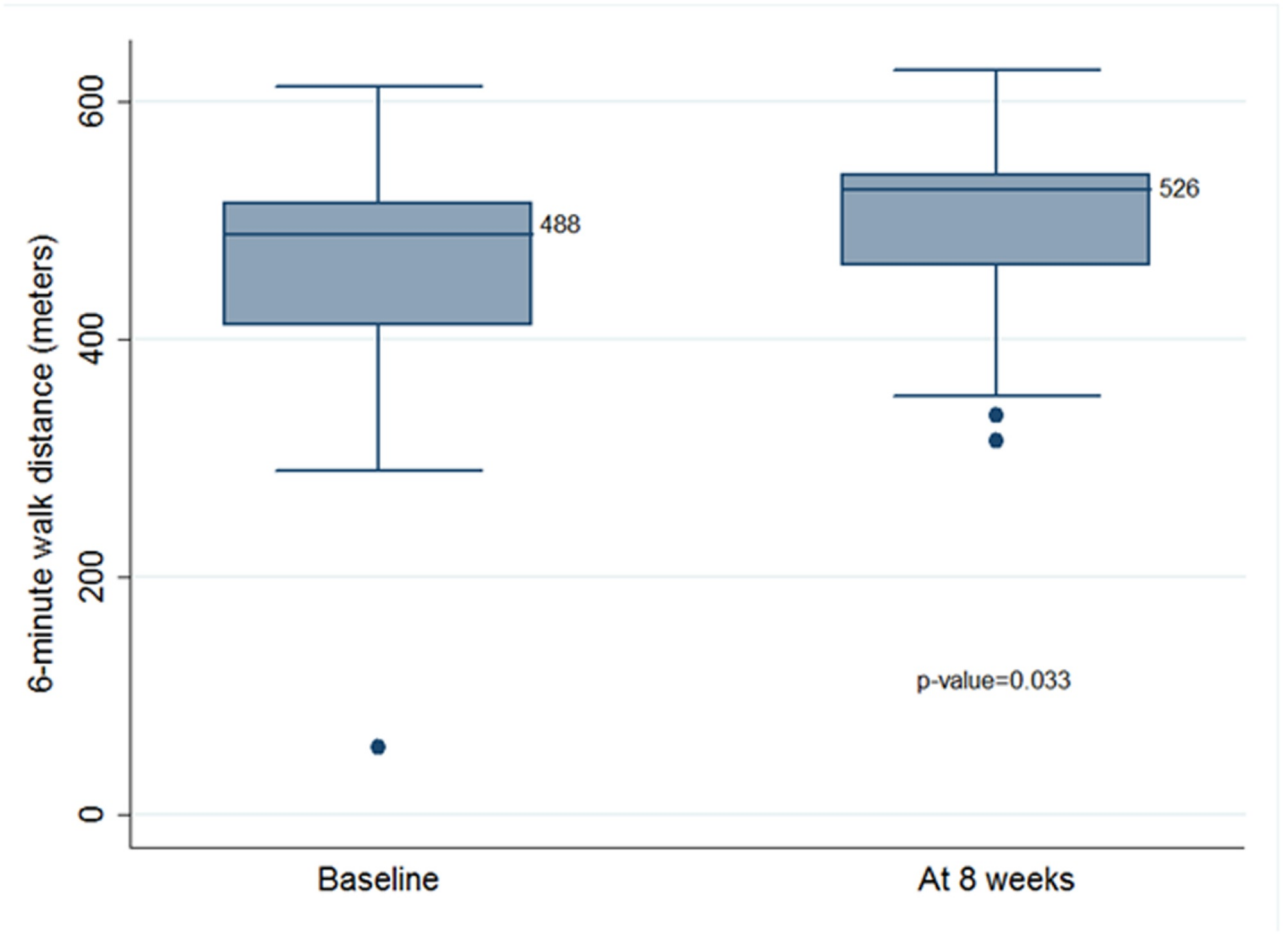
Six-minute walk distance.

Inflammatory markers also did not show any significant change upon completion of PR [TGF-β1 (1.3 vs 1.43, p-value 0.91) and hs-CRP (4.3 vs 2.3, p-value 0.21)]. Similarly, there was no significant improvement in muscle mass (MTCSA_CT_ 9264 mm^2^ vs 9687.44 mm^2^, p-value 0.30). However, we saw a significant improvement in the 6-MWD with eight weeks of PR (488 meters at baseline vs 526 meters post PR intervention, p-value 0.033) (Figure 2).

HRQL measure/dyspnea score, CRQ, also showed a significant improvement (17.21 at baseline vs 18.96 post PR intervention, p-value 0.025) however, the MMRC score showed no significant change (2.1 at baseline vs 2.0 post PR intervention, p-value 0.58).

## Discussion

pTB is caused by Mycobacterium tuberculosis when droplet nuclei laden with bacilli, from people with active pTB, are inhaled. In more than 90% of people infected with Mycobacterium tuberculosis, the pathogen is contained as an asymptomatic latent infection. The risk of active disease is estimated to be approximately 5% in 18 months after initial infection and then approximately 5% for the remaining lifetime [13]. Given the extent of the disease and success of treatment, the number of living tuberculosis survivors is increasing worldwide.

Histopathologic findings in TB include caseating granuloma, liquefaction and cavity formation. These pathologies may lead to bronchial and parenchymal structural changes, including broncho-vascular distortion, bronchiectasis, emphysematous changes, and fibrotic bands [7]. While these changes remain after initial cure, subsequent follow-up of these patients is not recommended per the guidelines [14]. Besides the structural changes, studies have shown residual lung function impairment in post-tuberculosis patients. Variable patterns (obstructive, restrictive or mixed) and severity of pulmonary function impairment have been described [15,16]. It is thus evident that pulmonary tuberculosis is an important, mostly under-recognized contributor of chronic lung disease, particularly COPD and bronchiectasis [17].

It is of interest whether strategies used in COPD such as vaccination, would be effective in preventing morbidity and mortality in patients suffering from chronic lung impairment from tuberculosis. PR is a comprehensive intervention that includes exercise training, education, and behavior change, and is indicated in all individuals with chronic lung impairment, be it from COPD, interstitial lung disease, bronchiectasis, asthma, cystic ﬁbrosis, lung transplantation, lung cancer or pulmonary hypertension [8]. There is growing evidence that suggests the benefit of PR in individuals with CLIPTB [11,12]. In our study, we did not find a significant difference in PFTs with ongoing PR although both FEV1 (1.02 liters at baseline and 1.07 liters at eight weeks) and FVC (1.76 liters at baseline and 1.81 liters at eight weeks) showed some improvement (Table 2). Other studies have reported similar findings [18].

In addition to abnormal histology and lung function tests, pTB is also associated with increased levels of transforming growth factor β1 (TGF-β1) in the serum. TGF-β1 is a peptide secreted by a variety of cells in the body and is known to be the central mediator of fibrogenesis [19]. Ameglio et al. reported that the extent of fibrosis in TB-infected lungs was directly proportional to the TGF-β1 levels. They concluded that TGF-β1 levels might be analyzed as a prognostic index for fibrotic evolution in adequately treated TB patients [20]. As increased fibrosis is associated with increased incidence of bronchiectasis, it could be postulated that TGF- β1 levels may correlate with incidence of CLIPTB. hs-CRP is another serum marker that is elevated in acute pTB and has been shown to decrease with treatment. Although no studies have looked into hs-CRP levels in CLIPTB patients, there is abundant literature showing direct correlation between hs-CRP levels and dyspnea, quality of life and degree of airway obstruction in COPD [21,22]. In our study, we found a non-significant decrease in these serum markers with PR (Table 2).

TB also leads to wasting from a combination of a lack of appetite and the altered metabolism associated with the inflammatory processes and immune responses. Wasting involves the loss of both fat and lean tissue and the decreased muscle mass contributes to the fatigue [23]. Measurement of body weight or body mass index (BMI) does not accurately reflect changes in body composition in these patients [24]. Studies in patients with COPD have shown MTCSA_CT_ to be a better predictor of mortality than BMI [25]. We extrapolated the use of MTCSA_CT_ to our CLIPTB patients and measured MTCSA_CT_ at baseline and eight weeks while undergoing PR. We found a non-significant increase in the MTCSA_CT_ with PR (9222.3 mm^2^ vs 9356.67 mm^2^ at eight weeks). Wasting also leads to a decline in exercise capacity. As our primary outcome, we assessed limitation in exercise capacity by 6-MWD, which has been widely used in objective assessment of patients with chronic lung diseases [26]. In our study, we found a statistically significant increase in the 6-MWD with PR (488 meters at baseline vs 526 meters post PR intervention) (Table 2). These findings are similar to findings from other studies [11,12,18].

It has been recognized that physiological measures do not necessarily relate to function, and that functional outcomes need to be measured independently [27]. Measuring HRQL is one method of evaluating functional outcomes. The MMRC and CRQ are two of the most widely used and optimal methods to assess HRQL in patients with chronic lung diseases [27-29]. In our study, we found a significant improvement in CRQ (17.21 at baseline and 18.96 at eight weeks) with PR. MMRC (2.10 at baseline and 2 at eight weeks) showed a non-significant improvement.

The six-minute walk distance is a significant measure for patients in daily life as it is a measure of sub-maximal exertion, the level of exertion people function at, for most hours every day. Being able to walk 38 more meters without getting short of breath (as we found in our study) is quite significant from the patient’s perspective. At the same time, improvement in dyspnea score (CRQ) with PR is quite relevant. If patients feel less symptomatic with their level of exertion, it reduces their anxiety when taking up tasks such as walking around the block to do groceries, which significantly improves their HRQL. Improvement in dyspnea score and 6-MWD make a strong argument to use PR in post-tuberculosis patients.

Being efficacious and cost-effective make PR a very attractive treatment strategy for CLIPTB patients. There is a need to revise the recommendations for follow-up of successfully treated pTB patients, to enable early detection and treatment of chronic lung impairment. Estimation of the burden of chronic lung impairment after adequate treatment for pTB by collecting more data would be helpful in guiding policy as well. It is of note that symptoms from chronic lung impairment do not present until FEV1 has fallen to ~50% of normal values and thus significant lung damage has already occurred. Thus a symptoms-based screening strategy for surveillance of chronic lung impairment following tuberculosis would be ineffective. Early identification and management of chronic lung impairment in these patients could improve morbidity and mortality. In the future, randomized controlled trials are needed to evaluate the effect of pulmonary rehabilitation on inflammatory markers in both acute tuberculosis patients as well as patients with CLIPTB.

## Conclusions

Our study shows that pulmonary rehabilitation is an effective intervention in post-tuberculosis patients. Appropriate utilization of pulmonary rehabilitation in this select population will improve the functional status and quality of life.

## Appendices

Supplement 1

The following investigations were done in all patients to ensure adherence to inclusion and exclusion criteria:

Complete blood count (CBC), comprehensive metabolic panel (CMP)

Chest X-ray (posteroanterior-view)

Electrocardiography (ECG)

Sputum for organisms: Gram stain, Ziehl-Neelsen stain, culture & sensitivity

Supplement 2

Pulmonary Function Testing

Spirometry was performed on a computerized apparatus- Benchmark (P. K. Morgan and Co. Ltd. Chatham, Kent England). Patients were instructed not to take any bronchodilators for six hours and oral theophylline for at least 24 hours prior to test. Maximal expiratory flow volume loops were obtained on a rolling seal spirometer. Reversibility was tested 20 minutes after the administration of 200 μg of inhaled salbutamol. At least three acceptable and repeatable maneuvers were obtained as recommended by the American Thoracic Society (ATS). Forced Vital Capacity (FVC), Forced Expiratory Volume in 1 sec (FEV1), FEV1/FVC% were measured using the selection criteria of ATS.

Measurement of Inflammatory Markers

Specimen Collection and Preparation

The specimen used for measuring various parameters was serum. Blood was collected using standard venepuncture technique. The blood was collected in a plain red top venepuncture tube without adding additives or anti-coagulants. Blood was then allowed to clot and samples were centrifuged at approximately 1000 x g for 10 minutes to separate serum from the cells. Serum samples were collected and frozen at -20°C. The stored samples were brought down to room temperature before using it for analysis of various parameters.

High Sensitivity C-reactive Protein (hs-CRP)

Preparation of Patient Serum

Patient serum was diluted 100 fold prior to use. 5 μl of serum was mixed with 495 μl of sample diluent.

Procedure

10 μl of hs-CRP standards, diluted patient serum were put into appropriate wells. 100 μl of hs-CRP enzyme conjugate reagent was added to each well and mixed well for 30 seconds. Samples were incubated at room temperature (18-25°C) for 45 minutes. Incubated mixture was removed by flicking the plate contents into a waste container. Microtiter wells were thoroughly rinsed and flicked five times with distilled water. The wells were striked down sharply onto an absorbent paper to remove all the residual water droplets. 100 μl of 3,3',5,5'-tetramethylbenzidine (TMB) solution was added to each well. The solution in each well was mixed for five seconds and incubated at room temperature for 20 minutes. 100μl of stop solution was added to stop the reaction and again mixed for 30 seconds until the blue color changes to yellow color. Absorbance was read at 450 nm with a microtiter well reader within 15 minutes. The minimum detectable level was 0.1 mg/L.

Transforming Growth Factor-β (TGF-β)

TGF-β was determined by commercial RayBio Human TGF-β2 ELISA (Enzyme-linked immunosorbent assay) kit. It is an in vitro enzyme-linked immunosorbent assay for the quantitative measurement of human TGF-β2 in serum, plasma, cell culture supernatants and urine. This assay employs an antibody specific for human TGF-β2 coated on a 96-well plate. Standards and samples are pipetted into the wells and TGF-β2 present in a sample is bound to the wells by the immobilized antibody. The wells are washed and biotinylated antihuman TGF-β2 antibody is added. After washing away unbound biotinylated antibody, Horseradish Peroxidase (HRP)-conjugated streptavidin is pipetted to the wells. The wells are again washed, a TMB substrate solution is added to the wells and color develops in proportion to the amount of TGF-β2 bound. The Stop Solution changes the color from blue to yellow, and the intensity of the color is measured at 450 nm.

Mid-thigh Muscle Cross-sectional Area by Computed Tomography (MTCSA_CT_)

A computed tomography of the right and left thigh, halfway between the pubic symphysis and the inferior condyle of the femur, was performed using a third-generation scanner. Each image was 10-mm thick and was taken at 120 KV and 200 mA with a scanning time of one second while the subject was lying in the supine position. The thigh muscle cross-sectional area (CSA) was obtained by measuring the surface area of the tissue with a density of 40 to 100 Hounsfield units (HU). The MTCSA_CT_ was calculated by taking average for right and left thighs.

Six-minute Walk Test (6-MWT)

Six-minute walk test was performed on a flat, straight, enclosed corridor with a hard surface as per the ATS guidelines. The walking course was 40 m in length and the length of the corridor was marked every 3 m and both the ends of the track were marked with cones. Patients were advised to take their regular medications and inhalers, to refrain from taking food, coffee or tea two hours prior to test and to wear clothes and shoes that were comfortable for walking. The patients were rested in a chair, located near the starting position for ten minutes before the test started. During this time patient’s baseline pulse rate, blood pressure, oxygen saturation, respiratory rate, grade of breathlessness and leg fatigue were recorded. Patients were instructed to walk as far as they can for six minutes. Standard instructions and encouragement were given to the patients. At the end of six minutes patients were asked to stop and their pulse rate, blood pressure, oxygen saturation, respiratory rate, grade of breathlessness and leg fatigue were recorded. The distance covered in six minutes was recorded in meters. To avoid inter-observer variability all tests were done by the same investigator.

Modified Medical Research Council Dyspnea Scale: Grade

0   “Patient only gets breathless with strenuous exercise”

1    “Patient gets short of breath when hurrying on the level or walking up a slight hill”

2    “Patient walks slower than people of the same age on the level because of breathlessness or has to stop for breath when walking at his own pace on the level”

3    “Patient stops for breath after walking about 100 yards or after a few minutes on the level”

4    “Patient is too breathless to leave the house” or “Patient is breathless when dressing”

Chronic Respiratory Disease Questionnaire (CRQ)

Developed by researchers at McMaster University, the Chronic Respiratory Questionnaire (CRQ) is a validated and reliable tool, widely used to measure health-related quality of life in patients with chronic airflow limitations. The instrument consists of 20 questions scored on a seven-point Likert-type scale in four domains: dyspnea, fatigue, emotional function and mastery.
